# Ecological Momentary Assessments and Automated Time Series Analysis to Promote Tailored Health Care: A Proof-of-Principle Study

**DOI:** 10.2196/resprot.4000

**Published:** 2015-08-07

**Authors:** Lian van der Krieke, Ando C Emerencia, Elisabeth H Bos, Judith GM Rosmalen, Harriëtte Riese, Marco Aiello, Sjoerd Sytema, Peter de Jonge

**Affiliations:** ^1^ University of Groningen University Medical Center Groningen University Center for Psychiatry Groningen Netherlands; ^2^ University of Groningen Johann Bernoulli Institute for Mathematics and Computer Science Groningen Netherlands

**Keywords:** ecological momentary assessment, time series analysis, vector autoregressive modeling, Web-based, dynamic effects, automatization, tailored treatment

## Abstract

**Background:**

Health promotion can be tailored by combining ecological momentary assessments (EMA) with time series analysis. This combined method allows for studying the temporal order of dynamic relationships among variables, which may provide concrete indications for intervention. However, application of this method in health care practice is hampered because analyses are conducted manually and advanced statistical expertise is required.

**Objective:**

This study aims to show how this limitation can be overcome by introducing automated vector autoregressive modeling (VAR) of EMA data and to evaluate its feasibility through comparisons with results of previously published manual analyses.

**Methods:**

We developed a Web-based open source application, called AutoVAR, which automates time series analyses of EMA data and provides output that is intended to be interpretable by nonexperts. The statistical technique we used was VAR. AutoVAR tests and evaluates all possible VAR models within a given combinatorial search space and summarizes their results, thereby replacing the researcher’s tasks of conducting the analysis, making an informed selection of models, and choosing the best model. We compared the output of AutoVAR to the output of a previously published manual analysis (n=4).

**Results:**

An illustrative example consisting of 4 analyses was provided. Compared to the manual output, the AutoVAR output presents similar model characteristics and statistical results in terms of the Akaike information criterion, the Bayesian information criterion, and the test statistic of the Granger causality test.

**Conclusions:**

Results suggest that automated analysis and interpretation of times series is feasible. Compared to a manual procedure, the automated procedure is more robust and can save days of time. These findings may pave the way for using time series analysis for health promotion on a larger scale. AutoVAR was evaluated using the results of a previously conducted manual analysis. Analysis of additional datasets is needed in order to validate and refine the application for general use.

## Introduction

### Person-Centered Research and the Idiographic Approach

Evidence-based treatment guidelines in health care are predominantly based on nomothetic, group-based research. Samples of patients are investigated to find general laws of symptomatology and functioning, which are then generalized to all individual members of the investigated population [1,2]. Several authors have criticized the dominance of this approach [3-6], which leads to knowledge that is “true on average” [2]. Although nomothetic research is useful to study variability between patients in a sample, the results do not necessarily generalize to individual patients. In fact, between-persons and within-person associations can diverge in both magnitude and direction [6]. In a study investigating the occurrence of desirable and undesirable events in the daily life of individuals with chronic pain, Tennen and Affleck [6] showed that, on average, there was a moderate positive association between desirable and undesirable events (*r*=0.50), indicating that patients experiencing more desirable events (relative to other people), also experienced more undesirable events. However, the within-person correlations showed an inverse relationship (mean *r*=-0.25), indicating that on days that patients reported more desirable events, they experienced fewer undesirable events. Another study focusing on personality traits in the general population shows that the Big Five factor structure, which resulted from a between-person analysis, could not be generalized to individuals. Within-person analysis showed differences between persons in both the number of factors and in how the factors related to the personality items [5]. The above examples illustrate that outcomes of nomothetic research need not be valid for individuals, as they tend to relate to what Gordon Allport in 1937 called “a nonexistent average individual” [7]. According to Allport, researchers should put more emphasis on the unique patterns within individuals over time. This is what he named the “idiographic approach.”

Allport was an early advocate of the idiographic, individual-based approach. In the 1960s and 1970s, the enthusiasm for idiographic research diminished. It was qualified as unscientific [8] and unrealistic [9], as there were no adequate methods for carrying out quantitative idiographic research [10]. At that time, idiographic research mainly referred to case study-based qualitative research. However, in the last 2 decades, new quantitative methods have been developed to perform idiographic research and researchers took up Allport’s ideas again, calling for a new person-centered approach in health research [3,5,6]. One of the most promising research methods that can be used to employ idiographic research is ecological momentary assessment (EMA), also called experience sampling method or diary methods [11]. EMA is aimed at repeatedly assessing experiences, activities, and physiological parameters, once or multiple times a day, and is typically characterized by real-time data collection in a natural setting [3]. EMA data can be analyzed at the group level by, for instance, multilevel analysis [12]. However, a more recent development is time series analysis, which allows for the analysis of EMA data on an individual level (level of the idios). The combination of EMA with time series analysis, which we refer to as “intensive time series design,” has recently brought the idiographic approach back to life.

### Intensive Time Series Designs in Health Research

A number of research examples can be found in which intensive time series designs are used to map the mental and physical functioning of individual people [13-18]. For instance, Bouchard et al [13] investigated the temporal relationships between dysfunctional beliefs, self-efficacy, and panic apprehension in a diary study of 12 patients suffering from anxiety. Multivariate time series analysis identified substantial heterogeneity between the patients in the dynamic associations between variables. In 3 patients, changes in panic apprehension were predicted by changes in dysfunctional beliefs, in 6 patients they were predicted by changes in self-efficacy, and in 2 patients they were predicted by both changes in dysfunctional beliefs and self-efficacy.

In another study, Rosmalen et al [18] used time series analysis to investigate the causal direction of associations between physical activity and depression in 4 patients who had experienced a myocardial infarction. They found that in 2 patients, depression predicted physical inactivity; in 1 patient, physical inactivity predicted depression; and in another patient, only a cross-sectional association between depression and physical inactivity was found. These results could be translated into concrete indications for treatment advice. For 1 patient, 1.5 hours of sports every 4 days led to a desirable degree of decrease in depressive symptoms, whereas for the other patients physical activity did not have beneficial effects on depression. These 2 studies indicate the potential of EMA combined with time series analysis for health care practice. The identification of individual patterns of symptoms, behaviors, and experiences sheds light on the most important functional and dysfunctional dynamics of a given person, providing concrete indications for tailored treatment advice [18].

### Gap Between Research and Health Care Practice

Despite the promising examples described above, there still is a significant gap between the research context in which intensive time series analysis is experimented with and health care practice in which individual patients may profit from its results. An important challenge is the substantial burden that data collection and processing puts on patients and researchers. Patients have to complete at least 50 assessments, and preferably even more [19]. Researchers have to be experienced in advanced time series methodology, which they have to apply at an individual level, for each person separately. This has led some researchers to conclude that the idiographic approach is too time-consuming and too expensive for implementation on a large scale [20].

Intensive time series analysis can only be applied in daily care practice when certain requirements are met. First, data collection and data management should be standardized to some extent, as to enable professionals and patients to select relevant assessment domains from a prespecified set of measures. This is to prevent a situation in which intensive time series data collection needs to be built from scratch for every individual patient. Second, to deploy intensive time series in the course of a treatment process, as a diagnostic means, or as a method to evaluate treatment effects, time series data need to be available real-time so that the outcomes can be used immediately. Third, it should be possible to conduct a reliable analysis of time series data, without extensive statistical training. Fourth, professionals and patients should be able to interpret the output of intensive time series and to understand how the results relate to their particular care context.

The latter 2 conditions, which allow for a situation in which the researcher becomes superfluous, may be the hardest and most fundamental conditions to meet. So far, analysis of time series data has always required advanced statistical expertise, including extensive knowledge of the statistical procedures and a high level of experience.

### Statistical Modeling of Time Series

There are several forms of time series data. Time series can be event-based, in which the assessments follow a specific event, or time-based in which the assessments are performed at specific time points. Moreover, time-based assessments can be conducted either at fixed or random moments. Each method has its own purposes. If data is collected at fixed moments, with equidistant intervals in between time points, temporal dynamics between variables can be analyzed by a method such as vector autoregressive modeling (VAR) [19,21,22].

The “vector” term in vector autoregressive modeling refers to the multivariate character, which is an extension of the single variable autoregressive model. VAR models consist of a set of regression equations in which all variables are treated as endogenous variables, meaning that they function as both outcome and predictor. VAR analysis can be conducted without a prior hypothesis about the direction of the association between variables. A statistical test called the “Granger causality test” can be used to examine whether the lagged values of one variable x are useful in the prediction of values of another variable y. If so, it is said that variable x *Granger-causes* variable y [23]. VAR analysis can thus elucidate dynamic relationships between 2 or more variables, providing an impression of putative causal associations. The identification of these dynamic relationships, in turn, paves the way for unveiling detailed and patient-specific patterns of symptoms or experiences, their triggers, and their effects on functioning. An extensive description of the VAR technique can be found elsewhere [19,21,22]. At this point we should note that in the practice of EMA assessments, the distance between two consecutive time points often is not equal. In these cases, the raw time series data would not meet the VAR modeling assumption of equidistant time intervals. The EMA data can, however, be preprocessed such that they do meet this assumption. One such way of reprocessing is to use spline smoothing, followed by resampling at equal sampling intervals [24,25].

In the VAR modeling process, researchers are broadly faced with 2 main tasks, namely (1) to build statistical models and conduct a reliable, iterative analysis to evaluate the validity of these models and (2) to choose the best model with which they can work. The first task is predominantly a statistical one. Although the researcher has to make some choices, such as which variables to include in the VAR and the maximum lag length (ie, the maximum number of previous observations that contain relevant information for estimating the current observations), the biggest part of this task consists of statistical analysis conducted with predefined tests. By means of residual diagnostics, the models are checked for assumptions of stability, “white noise” (ie, no residual autocorrelation), homoscedasticity, and normality based on which valid models can be selected. The second task is less statistical. Choosing the “best” model out of all valid models mostly is an informed choice of content. It is based on a combination of statistical parameters (eg, model selection criteria like the Akaike information criterion (AIC) or the Bayesian information criterion (BIC)) theoretical assumptions about the data, and common sense. The researcher plays a crucial role here.

### Aim

Quantitative idiographic assessment has shown to be promising, but application of this method in health care practice is hampered because analyses are conducted manually and advanced statistical expertise is required. This study aims to show how this limitation can be overcome by introducing innovative technology.

We provide a proof-of-principle that might bring idiographic assessments closer to health care practice by automating analytical processes. We developed a Web-based application, called AutoVAR, which automates time series analyses of EMA data and provides output that is intended to be interpretable by nonexperts. We report on our experiences with the program in re-analyzing a set of time series data.

## Methods

### Patients and Measures

To evaluate the outcomes of our automated analysis, we reanalyzed data that were previously analyzed in a manual analysis in a study by Rosmalen et al [18]. This data was obtained from 5 middle-aged (55-59 years old) Caucasian male patients suffering from post-myocardial infarction, recruited from screening for a psychoeducational prevention module (PEP) at the Máxima Medical Center in Eindhoven-Veldhoven, the Netherlands. The PEP module focuses on regaining emotional stability and dealing with cardiac disease as part of a cardiac rehabilitation program. Patients were considered eligible for study participation if they had a score of 10 or higher on the Beck Depression Inventory (BDI) [26], meaning that they suffered from mild to moderate depressive symptoms. The BDI is a self-report questionnaire assessing depressive symptoms in a reliable and valid manner [27]. The questionnaire addresses both cognitive and somatic depressive symptoms during the past week, such as hopelessness, guilt, fatigue, and weight changes. The BDI has 21 items, scored on a scale ranging from 0 to 3. Exclusion criteria for the study were significant cognitive impairments, life-threatening diseases, and severe problems with physical activity. Written informed consent was obtained from all patients. The study was approved of by the Medical Ethical Committee for mental health institutions in the Netherlands. Data collection took place in the first semester of 2010.

Patients were asked to complete daily measures of depressive symptoms and physical activity every evening, during a period of 3 months. Depressive symptoms were measured with the depression module of the Patient Health Questionnaire [28], which was adapted for daily use. The Patient Health Questionnaire includes 9 items assessing depressive symptoms based on the DSM-IV criteria for major depressive disorder. The items were rated on a 4-point scale ranging from 0 to 3. The sum score (0-27) was used as a measure of depression severity. Level of physical activity was measured by 7 items describing physical activities (eg, commuting activities, work activities, household activities, sports, and other leisure activities), of which patients had to report the amount of time in minutes they had spent on them [18]. The total daily amount of time being physically active was used in the analysis.

To encourage compliance to the daily assessments, patients were promised that they would be provided with a personal report of the assessments results after completion of the assessments. They were also offered a small gift certificate of €25. During the study period, one patient dropped out after 2 weeks because he was too busy at work and could not manage to complete the daily assessments. This patient was not included in the analysis.

### Automated Time Series Analysis With AutoVAR

**Figure 1:**
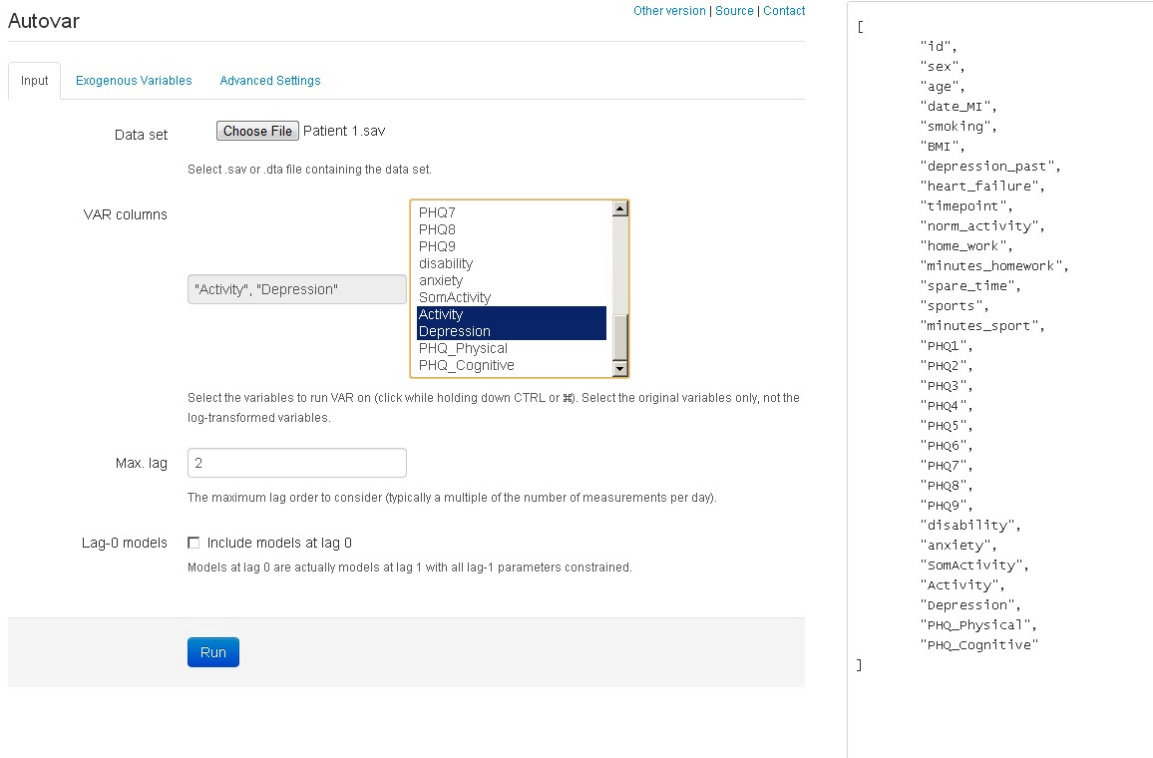
AutoVAR screenshot.

Our starting point was the study by Rosmalen et al [18]. We aimed to investigate whether the complex time series analysis using VAR modeling, which was conducted manually in the Rosmalen study, could be automated. To automate the analysis processes performed by Rosmalen et al, one of the authors (AE) developed an open source R package that includes a front-end Web application. This application reads raw data in an SPSS or STATA file and fits the data into VAR models. For the VAR modeling, the existing R package for VAR modeling is used [29]. In the new application, one can upload a data file, select variables for time series analysis, specify the maximum number of lags, and run the program (see Figure 1). For this paper, we selected the variables Activity and Depression from the Rosmalen dataset. The right column in Figure 1 shows all variables included in the dataset. Under the tab “Exogenous variables” one can add exogenous variables. Under the tab “Advanced settings” one can change settings (eg, change ordering from AIC to BIC scores).

AutoVAR is developed to take over those actions that in the manual analysis can only be conducted by a statistical expert. The solution that AutoVAR follows is to test all possible models within given restrictions and to summarize outcomes of all valid models. When the program is running, AutoVAR creates time plots for each selected variable, defines the possible VAR models, checks all models for validity, and finally presents all valid models. AutoVAR is freely accessible online and it is accompanied by documentation and a user example [30-32]. For a discussion of AutoVAR from a computing science perspective, see also Emerencia et al [33]. (We would like to note that the AutoVAR package is work in progress. We are currently working on improving the package’s functionality.)

The total number of possible VAR models is determined by the combinatorial search space. AutoVAR’s combinatorial search space is defined by multiple factors:

1. The lag length. The lag length refers to the maximum number of previous observations that contain relevant information for estimating the current observations. AutoVAR tests all lag lengths, up to a maximum set by the user by typing the number into the box “Max. lag.” In this paper, the maximum lag length was set to 2, following the procedure by Rosmalen et al [18]. In a manual analysis, a researcher tests those lag lengths that seem to make sense, based on theoretical plausibility, common sense, and lag length selection criteria (eg, the likelihood ratio test, final prediction error, Akaike information criterion, Hannan-Quinn information criterion, and the Bayesian information criterion).
2. Potential need for inclusion of a trend variable. AutoVAR checks whether a time series is stationary around a trend with the Phillips-Perron test [19]. If this test is significant, a trend term is added to the model as an exogenous variable. In a manual analysis, the presence of a trend variable is determined either by looking at the time plots and judging whether the mean of the time series changes over time or on the basis of a stability test (eg, the Eigen value stability condition [19]).
3. Potential need for inclusion of seasonal variables. AutoVAR checks whether seasonal variables should be included using dummies for the weekdays (if AutoVAR’s option “timestamps” is checked). AutoVAR evaluates, by default, every model twice. Once with and once without dummy variables for weekdays. In a manual analysis, dummy variables for weekdays are added when it seems to make sense, for instance when a lag of 7 is indicated by lag length selection criteria.
4. Potential presence of outliers. Outlier values that violate model assumptions are accounted for in AutoVAR and manual analyses by including a dummy variable as an exogenous variable (eg, 0/1). In AutoVAR, outliers are defined as values larger than 3.5, 3.0, or 2.5 standard deviations from the mean of the residuals. AutoVAR will first test a model without outliers; if this model is invalid, it will test a model with outliers that deviate 3.5 standard deviations from the mean of the residuals; if the model is still invalid, it will test a model with outliers that deviate 3.0 standard deviations. If this still yields no valid model, AutoVAR will stop, unless the option is checked to look for outliers of 2.5 standard deviations. In a manual analysis, the presence of outliers is determined by looking for extraordinary values in the time plots of the (residuals of the) variables and based on additional information provided by the patient.
5. Log transformation. AutoVAR constructs and calculates each model with and without log transformation. In a manual analysis, a researcher determines whether a log transformation is necessary based on a normality test, such as the Skewness-Kurtosis test [19]. If this test is significant, the residuals do not have a normal distribution. A log transformation is applied when this non-normality is caused by a skew to the right. This skew to the right can be determined by checking the histogram, the time plot, or the box plot of the residuals.
6. Potential need for constraints put to model parameters. Like the manual procedure, AutoVAR sets to “0” those parameters that do not significantly contribute to the model, starting with the parameter that has the highest *P*-value. After each constraint, the VAR model is rerun, until the chosen goodness-of-fit criterion (AIC or BIC) ceases to become smaller. In addition to the manual procedure, AutoVAR checks assumptions for stability, “white noise,” homoscedasticity, and normality after every constraint has been set (see also below).
7. Potential need for exogenous variables added to the model, based on additional patient information. Sometimes time plots show strange characteristics (eg, an unexpected increase in activity) that may be explained by external factors (eg, change of jobs). In AutoVAR, these external factors can be added to the model, by having the user select them as “additional exogenous variables.” In a manual analysis, the researcher adds additional exogenous variables to the model as part of the regular analysis procedure.

After each model is estimated, AutoVAR checks them for validity by means of an automated residual diagnostics procedure, in which 4 assumptions are tested. The stability assumption is checked by the eigenvalue stability condition, the “white noise” assumption by a Portmanteau test on the residuals, the homoscedasticity assumption by a Portmanteau test on the squares of the residuals, and the normality assumption by the Skewness-Kurtosis test (see [19]). All tests must be nonsignificant for all variables for AutoVAR to consider a model valid. If one of these tests indicates a violation of the model assumptions, the model is adjusted, reestimated, and reevaluated in an iterative model building process until all assumptions are met (or until meeting all assumptions appears impossible, meaning that no valid models can be found). This process is similar to the manual procedure.

The validity of models also plays a role in the total number of models that AutoVAR runs. Strictly speaking, AutoVAR does not run all possible models defined by the combinatorial search space, but only the nonredundant ones. Of all the models that AutoVAR considers, it filters out the redundant models prior to running the final model calculations. AutoVAR considers a model redundant when it is not needed for optimization of the data modeling. For instance, a valid model without modeled outliers makes a model with the exact same model specifications but with modeled outliers redundant. This is to say that AutoVAR always tries to fit the most simple model (eg, without outliers) to the data first and only resorts to more complex models (with outliers) when these simple models do not suffice (ie, when they invalidate one or more of the model assumptions). This procedure has consequences for the number of valid models that can be fitted to the data. If simple models do not suffice to fit the data, AutoVAR has to resort to more complex models and thus the total number of possible models increases. For instance, if a model without outliers is not valid, AutoVAR will widen the combinatorial search space to include models with outliers. As a result, the total number of valid model fits for complex models often will be higher than the total number of valid model fits for more simple models. Finally, for all valid models, AutoVAR calculates AIC and BIC scores. AutoVAR orders the valid models on ascending order of the best (ie, lowest) AIC or BIC score. If the ordering of models based on AIC scores differ from the ordering based on BIC scores, AutoVAR will present the ordering based on AIC by default. However, users have the option to change the ordering based on BIC score by checking a box on the Advanced Settings page. Results of Granger causality tests are summarized in an image.

The AutoVAR procedure deviates from the manual procedure in two important respects. First, AutoVAR tests all possible VAR models within a given combinatorial search space, whereas a researcher tests a selection of models based on statistical and theoretical considerations. Second, AutoVAR orders the valid models and presents all of them in a Granger causality image, whereas a researcher evaluates the models and chooses one “best” model.

### VAR Model

**Figure 2:**
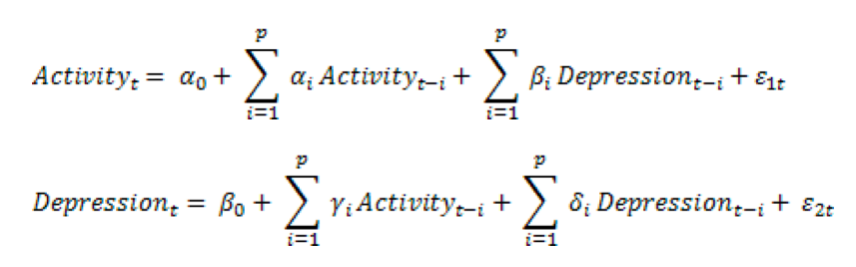
The endogenous variables for depression and physical activity used in this study.

The basic VAR model used in this study was the same model as the one used by Rosmalen et al. The model consists of a system of two endogenous variables, namely, depression and physical activity, which are shown in Figure 2 below.

In these equations *α*_i,_ *β*_*i*_, *ϒ*_*i*_ , and *δ*_*i*_ are the coefficients to be estimated, *p* is the number of lags considered in the system, and the ε_t_ is the stochastic error term. Each endogenous variable is made up of a constant, a regression coefficient determined by its own *p* lagged value, a regression coefficient determined by the *p* lagged value of the other variable, and a random error component. The error terms should be serially uncorrelated but can be contemporaneously correlated. Potential confounding factors can be accounted for by adding a control variable to the VAR model (not included in the formulas). This control variable is an exogenous variable, meaning that the variable can affect the model but cannot be affected by the model.

There are 4 main assumptions that need to be met for a VAR model to be valid: (1) the stability assumption requires that the VAR model is stable (ie, that it is stationary over time), (2) the “white noise” assumption requires a model to leave no autocorrelation in the residuals, (3) the homoscedasticity assumption requires homogeneity of variance over time, and (4) the normality assumption requires the residuals to be normally distributed.

In the Rosmalen et al study, the VAR analyses were performed in STATA 11 software, using the suite of VAR commands [34]. AutoVAR uses the existing R software package for VAR modeling [29].

## Results

### Output of AutoVAR

For patient 1 of the study by Rosmalen et al [18], AutoVAR generates the following output (see Table 1, first column). It provides a time plot of the activity and depression series, showing how activity and depression fluctuate over time. Furthermore, the textual output of AutoVAR summarizes the VAR model selection procedure. Forty-three VAR models out of 216 possible combinations were tested (19.9%). In the combinatorial search space, 216 is the maximum number of models that can be created, with a maximum lag length set on 2 (ie, 2 days). A total of 173 models were not tested due to redundancy. Of the 43 models tested, 2 of them appeared to be valid, meaning that they met the assumptions of stability, “white noise,” homoscedasticity, and normality. Both models indicated that physical activity Granger-caused depression and that the sign of the association was negative. In AutoVAR, the best model was presented at the top, with an AIC of 631.22 and a BIC of 655.41.

**Table 1:** Comparison of AutoVAR output versus manual analysis output.

		Autovar analysis	Manual analysis
Patient 1 (T^a^= 83)	Granger causality Wald test	Increase activity → decrease depression (*P*=.03)	Increase activity → decrease depression (*P*=.03)
	Lag length	2	2
	Trend variable included	No	No
	Weekday dummies included	No	No
	Outlier variables	Outlier dummies for day 4 (Depression) and day 13 (Activity)	Outlier dummies for day 4 (Depression) and day 13 (Activity)
	Log transformation	No	No
	BIC	655.41	655.89
	AIC	631.22	631.70
Patient 2 (T=63)	Granger causality Wald test	Not significant	Not significant
	Lag length	1	1
	Trend variable included	No	No
	Weekday dummies included	No	No
	Outlier variables	Outlier dummy for day 12 (Depression)	Outlier dummy for day 12 (Depression)
	Log transformation	Yes	Yes
	BIC	390.07	386.15
	AIC	381.49	375.43
Patient 3 (T=63)	Granger causality Wald test	Increase depression → decrease activity (*P*<.001)	Increase depression → decrease activity (*P*<.001)
	Lag length	2	2
	Trend variable included	Yes	Yes
	Weekday dummies included	Yes	Yes
	Outlier variables	Outlier dummy for day 5 (Depression)	Outlier dummy for day 5 (Depression)
	Log transformation	No	No
	BIC	307.21	304.64
	AIC	275.06	283.21
Patient 4 (T=58)	Granger causality Wald test	Increase depression → decrease activity (*P*=.04)	Increase depression → decrease activity (*P*=.04)
	Lag length	1	1
	Trend variable included	No	No
	Weekday dummies included	No	No
	Outlier variables	Outlier dummy for day 27 (Depression)	Outlier dummy for day 27 (Depression)
	Log transformation	Yes	Log transformation yes
	BIC	398.59	398.59
	AIC	386.23	386.23

^a^T is the number of time points at which patients completed a measure.

**Figure 3:**
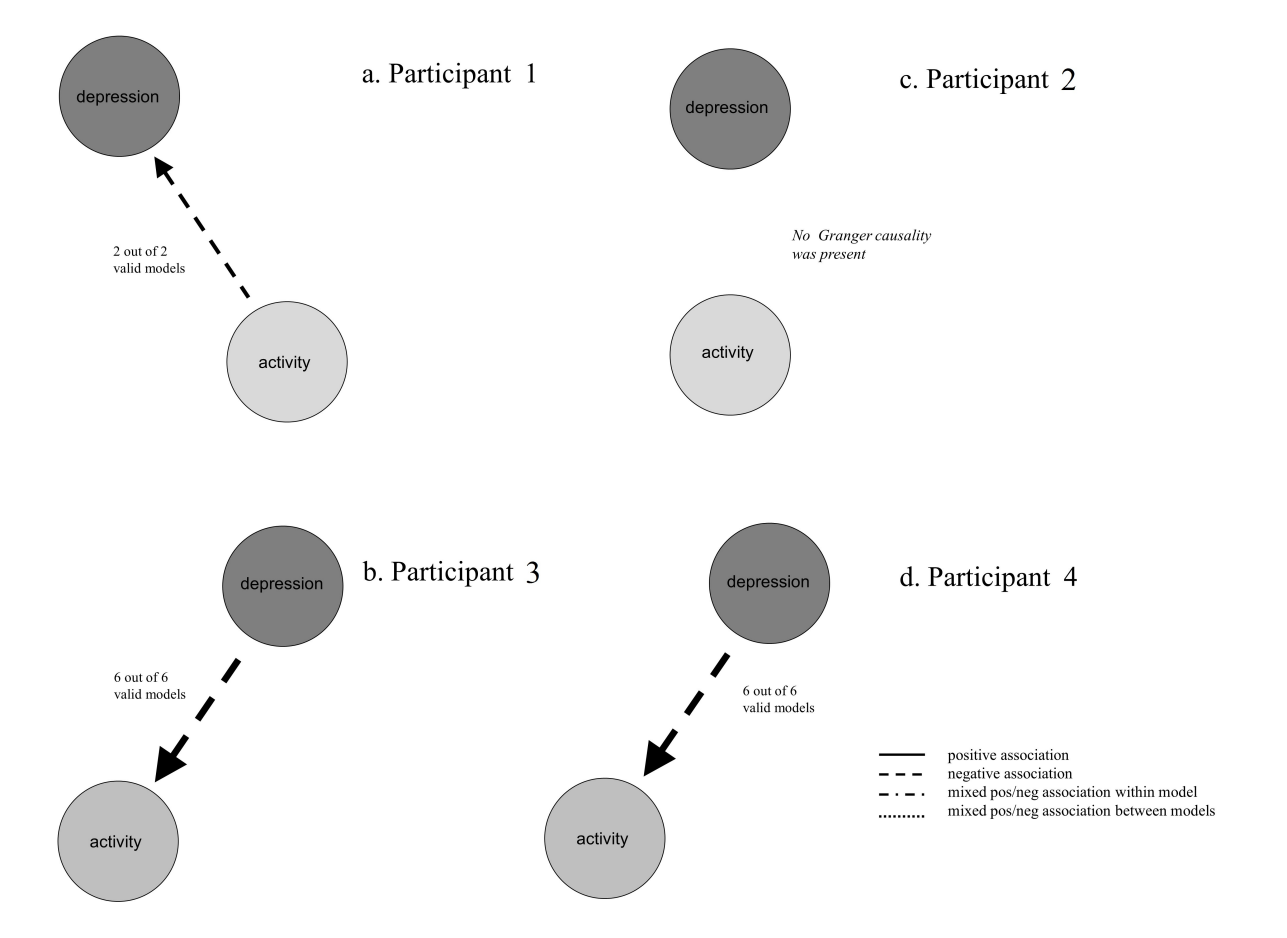
Granger causality plots.

The results of the Granger causality tests of all valid models are summarized visually, in a rather self-explanatory image in Figure 3. The thickness of the line connecting “Activity” with “Depression” indicates the proportion of valid models in which this Granger causal association was found (the thicker the line, the more models), which can be interpreted as the probability of the effect. The arrow shows the direction of the association. The line style (or, in AutoVAR, the color of the line) designates the sign of the association: continuous means a positive association, dashed with equal dashes equals a negative association, dashed with unequal dashes means a mixed positive and negative association within the model (ie, estimates show a positive and a negative sign, at different lags, within the model), dashed with points shows mixed positive and negative association among models (ie, some models show a positive, some a negative sign). From the first image in Figure 3, one can infer that for patient 1 inactivity is likely to Granger-cause an increase in depressive symptoms, whereas there is no indication that it is the other way around.

For the other 3 patients in the Rosmalen et al study, the Granger causality images generated by AutoVAR are also presented in Figure 3. For the data of patient 2, AutoVAR concludes “Granger causality summary: none.” The data of patient 2 did not show any Granger causal associations, meaning that no image could be created. For patients 3 and 4, the image shows that their depressive mood is likely to Granger-cause them to become physically inactive, whereas there is no indication that inactivity Granger-causes depression. These Granger causality images provide diagnostic information that can be used rather intuitively to guide tailored treatment decisions. If the time series data of patient 1 show that inactivity is likely to increase depressive symptoms, then it makes sense to advise this patient to become more active, as this may have a positive effect on his mood. In contrast, patients 3 and 4 probably would not benefit from this advice. Their personal network indicates that a depressive mood has an effect on physical activity instead of the other way around. In their case, the main target of intervention would be the depressive symptoms. These patients might therefore profit more from therapy targeting their depressive symptoms, such as pharmacotherapy or psychotherapy.

### Automated Analysis Compared to Manual Analysis

Comparing the output generated by AutoVAR to the outcomes resulting from the manual analysis described by Rosmalen et al, we found rather similar results in terms of model specification, model validity, information criteria, and Granger causality estimates (see Table 1). For patient 1, both methods found an optimal lag length of 2, included no trend or seasonal variables (weekday dummies), required no log transformation, and included the same two outlier variables. Furthermore, the top model in the ordering by AutoVAR (both AIC and BIC orders) matched the best model chosen in the manual analysis by Rosmalen et al, and both showed a significant negative Granger causal relationship between activity and depression.

##  Discussion

### Principal Findings

In this paper, we provided a potential solution to bridge the gap between the use of intensive time series analysis in research and health care practice by automating the analysis processes. Results suggest that automated time series analysis is feasible and that the output can be presented in an intuitive way. Automated analysis can make the role of the statistical interpretation less important and, as such, it saves a significant amount of time. Whereas AutoVAR generates results in a few seconds, manual analysis may take several days. Automated analytical procedures and accessible visual presentation of statistical outcomes might pave the way for health care professionals and patients to use methods such as EMA as an integral part of the treatment trajectory, without extensive training. As such, general treatment guidelines based on nomothetic research could be complemented by idiographic-based information. This may support health care professionals in taking a tailored treatment approach. Although the personal narrative of patients remains an important basis for tailor-made treatment, intensive time series assessments can add information that professionals are unable to see with the naked eye. EMA may be particularly valuable in those situations in which treatment trajectories have become stuck, when patients do not sufficiently benefit from treatment, and professionals do not know why. Furthermore, since completing EMA assessments can be quite an investment, an automated EMA approach may be especially suitable for settings in which patients receive long-term treatment for a chronic disease, such as depression or a heart disease in which controlling, instead of curing, is the main focus. The creation of a thorough and detailed patient profile of symptoms, behaviors, and experiences can help to shape the treatment toward individual needs.

Apart from EMA being an instrument to support professionals, we may also speculate that automated time series analysis provides opportunities for using EMA as part of self-management processes. If patients are able to analyze and interpret their own data, they may find it helpful to monitor themselves and map their symptoms or functioning in certain situations or periods. A promising perspective is sketched by Nikles et al [35] who conducted a study among patients with ADHD and osteoarthritis participating in idiographic research and found that the assessments led to increased knowledge and awareness of their condition, a better management of their bodily functions, and a sense of empowerment. We should note that if patients use EMA assessments for self-monitoring, they may change their behavior in response to their data, which implies that the resulting time series may no longer be stationary. However, these changes in behavior can be accounted for in the VAR model by adding a trend variable to the model.

### Strengths and Limitations

AutoVAR is promising, but the application needs further validation and refinement prior to implementation in health care practice. In this study, we applied AutoVAR to replicate the results of the manual analysis conducted by Rosmalen et al. Analysis of additional datasets is needed in order to validate the application for general use. Whereas the output of AutoVAR was rather similar to the manual output of the Rosmalen et al study and the most important output, namely the directions of the Granger causality relationships were identical, the model selection criteria (AIC and BIC) were not exactly the same in the different procedures. This may be due to differences in optimization algorithms in STATA versus R and therefore needs a more thorough scrutiny of discrepancies between the statistical packages in future research. An important question in this context is how to determine the validity of different procedures. In this paper, we compared automated analysis to manual analysis. Nevertheless, the manual analysis need not be the golden standard. The major advantage of a manual procedure is that a researcher can make informed decisions about the analysis process in a way that an application like AutoVAR can perhaps never do. These decisions are, however, subjective. They may depend on the researcher’s experience, preference, and “staying power.” As a consequence, valid time series models might be overlooked in a manual procedure. AutoVAR, in contrast, takes into account all possible models, thus following a more objective procedure. A limitation of this latter procedure is the risk of capitalization on chance. By testing many models, AutoVAR may generate more incidental findings. In the current version of AutoVAR, we tried to minimize this risk in 3 ways: (1) by not running redundant models, (2) by an extensive check of validity assumptions, and (3) by summarizing the results of the Granger causality tests in an image in which the thickness of the arrow indicates the probability of the effect.

The automated processes of the current version of AutoVAR need to be optimized. AutoVAR cannot yet handle missing data. VAR models can be processed with missing values, but this is suboptimal as this usually decreases the number of observations considerably, and thus decreases statistical power. Data collected from assessments completed at non-equidistant time intervals need to be preprocessed before AutoVAR can analyze them. There is as yet no functionality in AutoVAR to use spline smoothing and resampling of data. Moreover, AutoVAR currently functions most optimal when several settings are set manually. The lag length is one of these settings. AutoVAR also has several options that users can choose to check or leave blank, such as setting timestamps and adding additional exogenous variables based on patient information. These issues need to be solved before using automated analysis in health care practice. In addition, the user interface of AutoVAR has a rather technical look-and-feel and therefore needs a radical redesign to meet the criteria of user-friendliness for health care practice. We are currently working on an improved version of AutoVAR in which we will account for these issues.

One of the most important limitations of idiographic analyses compared to nomothetic analyses is their presumed limited generalizability. What holds for one individual is not necessarily true for another. Nevertheless, the question is whether this limitation needs to be overcome in the context of health care practice, for in this context the presumed weakness of idiographic research can also be considered one of its main strengths. If the main aim is to elucidate the specific temporal patterns of symptoms or experiences, and their triggers and effects on functioning within one specific patient, then the argument of generalizability to a larger population does not hold. The principal requirement for a meaningful use of intensive time series analysis as a supportive means in diagnostics and treatment of a specific individual is that the models selected provide a good description of the dynamic relationships in the EMA data registered by that very individual. Nevertheless, what remains is the issue of generalizability over time, within an individual. Whether the results of time series analysis need to be generalizable to the individual patient on multiple moments depends on the context. In those treatment contexts in which one is mainly interested in the temporal dynamics of variables in a specific time window, a single time series analysis may suffice and its results do not need to be generalizable to other points in time. Nevertheless, if one wants to generalize within one individual over time, for instance when the aim is to unveil the temporal dynamics of variables that are assumed to be rather stable, a second time series analysis is needed to confirm the explorative results of the first analysis.

Finally, instead of having nomothetic research replaced by idiographic research, the most ideal situation may be a combination of both. Gates et al [36] presented a procedure called Group Iterative Multiple Model Estimation that enables individual-level modeling while simultaneously identifying commonalities across individual models. Furthermore, time series analysis provides information about relationships between variables over time, but not about the meaning of mean levels of the variable values. To evaluate the level of variable values (eg, evaluating scores as falling into a clinical or nonclinical category), health care professionals and patients may profit from relating time series data to population-based norms.

### Implementation and Future Perspectives

The benefits of automated time series analysis can only be fully exploited when it is embedded in an “EMA-friendly” health care context. Just like the analysis and interpretation processes, the collection and management of data also need to be facilitated. This may best be realized by integrating time series assessments in the existing information technology infrastructure used by professionals and patients, such as systems for routine outcome monitoring (ROM). In the Netherlands, almost all mental health organizations use electronic ROM systems, which offer professionals and patients the opportunity to select and complete questionnaires and other measurements, of which the results are automatically presented in the electronic patient files. These systems were created for the mandatory yearly routine assessments among patients in which health care effects are examined. However, several systems have been extended with functionality for frequent and repeated assessments; for instance, by means of a diary app [37].

To facilitate intensive time series measurements, the electronic monitoring systems should include a specified set of reliable instruments that are appropriate for time series analysis of particular variables. From this set of instruments, health care professionals can select the relevant variables for specific patients. Time series diaries might also be automatically composed by having variables selected based on deviating scores on completed ROM measures. Time series measurements need not be restricted to self-report questionnaires. Current technological developments have given rise to smart and consumer-priced mobile devices measuring heart rate, activity, sleeping behavior, and so on. An increasing number of devices have a so-called open application programming interface, meaning that the data collected by these devices can be used by and be integrated into existing applications. Provided that they are validated, these devices can be excellent EMA data collectors. They often collect data automatically, so that minimal input is needed from the person who carries the device.

If patients are willing to participate in intensive time series measurement, they will have to deal with a long series of assessments. Motivation to complete the assessments is therefore crucial. A key element in motivating patients for EMA data collection is demonstrating to patients the personal and theoretical benefits EMA can have for them prior to the assessments [38]. Furthermore, during the assessment period, feedback on completed assessments may encourage patients to continue to next assessments. This feedback can consist of basic information about the percentage of successfully completed assessments or more advanced feedback about results obtained so far. Apart from the length, the repetitiveness of the assessments is an important obstacle in completing a time series [38]. A possible remedy to this problem may come from computerized adaptive testing and machine learning processes that can provide the basis for dynamic assessments, adapted to the individual [38].

Future studies should examine whether patients and care professionals are actually willing and able to use time series analysis in an individual care trajectory and how intensive time series analysis can best be integrated into the daily care practice. In addition, we need to investigate whether tailored treatment advice, based on the analysis, can improve clinical outcomes. After all, this is the ultimate test to determine the actual validity of intensive time series analysis for health care practice.

### Conclusions

In this paper, we have conducted a proof-of-principle study that has demonstrated the viability of a quantified idiographic approach in health care practice by using automated time series analysis. Compared to a manual procedure, the automated procedure is more robust and saves a significant amount of time. In addition, the output of automated time series analysis can be presented in an intuitive way. These findings may pave the way for health care professionals and those in need of care to use intensive time series analysis as an integral part of the treatment trajectory, without extensive statistical training.

## Abbreviations

AIC: Akaike information criterion
BIC: Bayesian information criterion
EMA: ecological momentary assessment
ROM: routine outcome monitoring
T: time points
VAR: vector autoregressive
